# Creation of a New Explosive Injury Equipment to Induce a Rabbit Animal Model of Closed Globe Blast Injury *via* Gas Shock

**DOI:** 10.3389/fmed.2021.749351

**Published:** 2021-09-23

**Authors:** Yuanyuan Liu, Tiantian Yang, Jinguo Yu, Mengxuan Li, Jianan Li, Hua Yan

**Affiliations:** Department of Ophthalmology, Tianjin Medical University General Hospital, Tianjin Medical University, Tianjin, China

**Keywords:** self-developed explosive injury equipment, rabbit animal model, closed globe blast injury, hyphema, retinal injury, corneal edema

## Abstract

To establish a rabbit animal model of closed globe blast injury with an application of self-developed explosive injury equipment, we tend to explore the anatomic and pathological changes of eyes under different gas pressure. The device comprises of high-pressure air source compression pump, air channel, and gas shock. There were 36 healthy bluish blue rabbits exposed to one of five blast pressures (500, 1,000, 1,500, 2,000, and 5,000 Kpa). Slit lamp microscope, B-mode ultrasonography, fundus photography, optical coherence tomography (OCT), and intraocular pressure (IOP) examination were performed at 0-, 1-, 3-, and 7-days post exposure, while gross histopathology was assessed with H&E stain at 7 days. The contralateral eyes and non-blast exposed rabbits were used as controls. Definitive evidence of closed globe blast injury was obtained. Corneal edema and hyphema were observed in the models under all pressures with no full-thickness globe injury, or lens rupture, as the severity was pressure independent. There was no obvious retinal abnormality on B ultrasound or OCT scan, while light vitreous hemorrhage, commotio retinae, and heavy retinal pigmentation presented on one eye, respectively, in the eyes exposed to 5,000 Kpa. Increased retinal thickness with disorganizations on the retinal ganglion cell (RGC) layer and RGC apoptosis in groups under higher pressure (>500 Kpa). IOP of injured eyes were statistically decreased at day 1 and 7 post injury (*p* < 0.05). Conclusively, the rabbit animal model induced by self-developed equipment could mimic the clinical features of closed ocular blast injury successfully that was feasible and easy to operate. This will be a new rabbit animal model for investigating mechanisms and new therapeutic interventions of closed globe blast injury in the future.

## Introduction

Mass casualties caused by blasts were common in war, bombing, or terrorism occurred around the world that have been documented in the Iraq War (2003), the Boston Marathon bombing (2013), and Tianjin Blast injury (2015), in which the rates of ocular injury were varied (1–3). Unlike other body parts covered with clothes, the eyes are more susceptible to blast injury due to lack of protection or spectacles wearing that can exacerbate the blast damage, especially in the military explosions where the soldiers wear chest and head protection. As reported, the injuries resulting from explosions are classified into four parts as followed: the primary injury caused by detonation wave itself, fragments propelled by the explosion (glass, dust, and masonry from exploded constructions), displacement of victims due to blast wind induced acceleration of the body, and thermal injury caused by tremendous and temporary heat produced by the explosion (4, 5). Hence, the ocular blast injury is combined and complicated eye damage that includes mechanical, chemical, and thermal damages, which can result in vision loss inordinately and even enucleation. Although there were ~80% of ocular injuries associated with blast fragmentation in military conflicts, the primary blast was predominantly accounted for the ocular blast injury (6). According to the published literatures, the categories of blast injuries were relatively feasible and well-studied aside from the primary blast, which might be due to the practical limitations of equipment and techniques involved in an experimental study.

As literature reported, a blast ocular injury can cause closed globe injury, open globe injury, and ocular foreign body when the explosive fragments retained. Cockerham's study indicated that the closed globe injury accounted for 43% of blast ocular injury, which was underestimated and neglected in the military survivors of blast injury (7). Blanch et al. illustrated types of closed globe injuries induced by blast in soldiers, such as corneal abrasions, corneal edema, iris, ciliary body contusion, hyphema, angle recession, cataract, commotio retinae, choroidal ruptures, vitreous hemorrhage, retinal hemorrhage, tears, traumatic retinal detachment, and traumatic optic neuropathy (8). Clinically, it is impossible to dissect out an ocular damage resulted from the primary blast wave alone in patients. Hence, it is necessary to establish an animal model of primary blast induce globe injury to reveal the underlying cellular and molecular mechanisms (9).

The literatures demonstrating the animal models that mimic progression of blast induced ocular injury have been published, in which various methods and mechanisms were applied. Rodents, such as mouse and rat were the most common animal species for the animal model development for low costs, multiple options for testing, and ease to obtain knock-out or overexpressed transgenic model for exploration of potential mechanisms (10), but with few samples obtained due to small size of the eyes. Enucleated porcine eyes were also used for their similarity to humans, especially in the anterior segments (11). Additionally, a computational model of a porcine eye was used to simulate the effects of the primary blast that was invented for mechanism exploring (12). However, a rabbit animal model was seldomly used. In those studies, devices for the blast generation were different, such as paintball guns, shock tubes, rifles, and small magnitude explosives at different levels of pressure (9, 13, 14). Although the corneal edema, retinal thickening, and optic neuropathy were observed in the experimental animal models, there are some disadvantages about the above devices and damage to the anterior segments were lacking. For example, the shock tubes occupy a large area, high cost, and are hard to control on the damaged location and degree; the paintball is dangerous to operate and cannot simulate the real explosion (15).

In this study, we invented a new gas shock generation device independently to create closed globe blast injury in rabbits by avoiding confounding injuries to the other parts of the body. We captured the progression and pathological changes of the blast induced globe injury in the rabbit animal model under different levels of gas pressure, which was identical to the veterans who suffered blast ocular injury in the clinic. Hence, our rabbit animal model may be an ideal model for the investigation of the mechanism and potential therapeutic strategy for human eyes.

## Materials and Methods

### Animals

There were 36 healthy blue rabbits of 3–6 months old and weighing 2–2.5 kg (male, purchased from Tianjin Yuda Lab, Tianjin, China) that were fed in Tianjin Medical University General Hospital Animal Room at controlled room temperature 20–27°C and humidity at 40–60%. Light in the animal house did not exceed 300 Iux with a 12 h light/dark cycle maintenance. This study was strictly in accordance with the standards of animal ethics and animal experiment regulations of the Animal Management Ethics Committee of Tianjin Medical University, Tianjin, China.

### Explosive Injury Equipment

**Figure 1:**
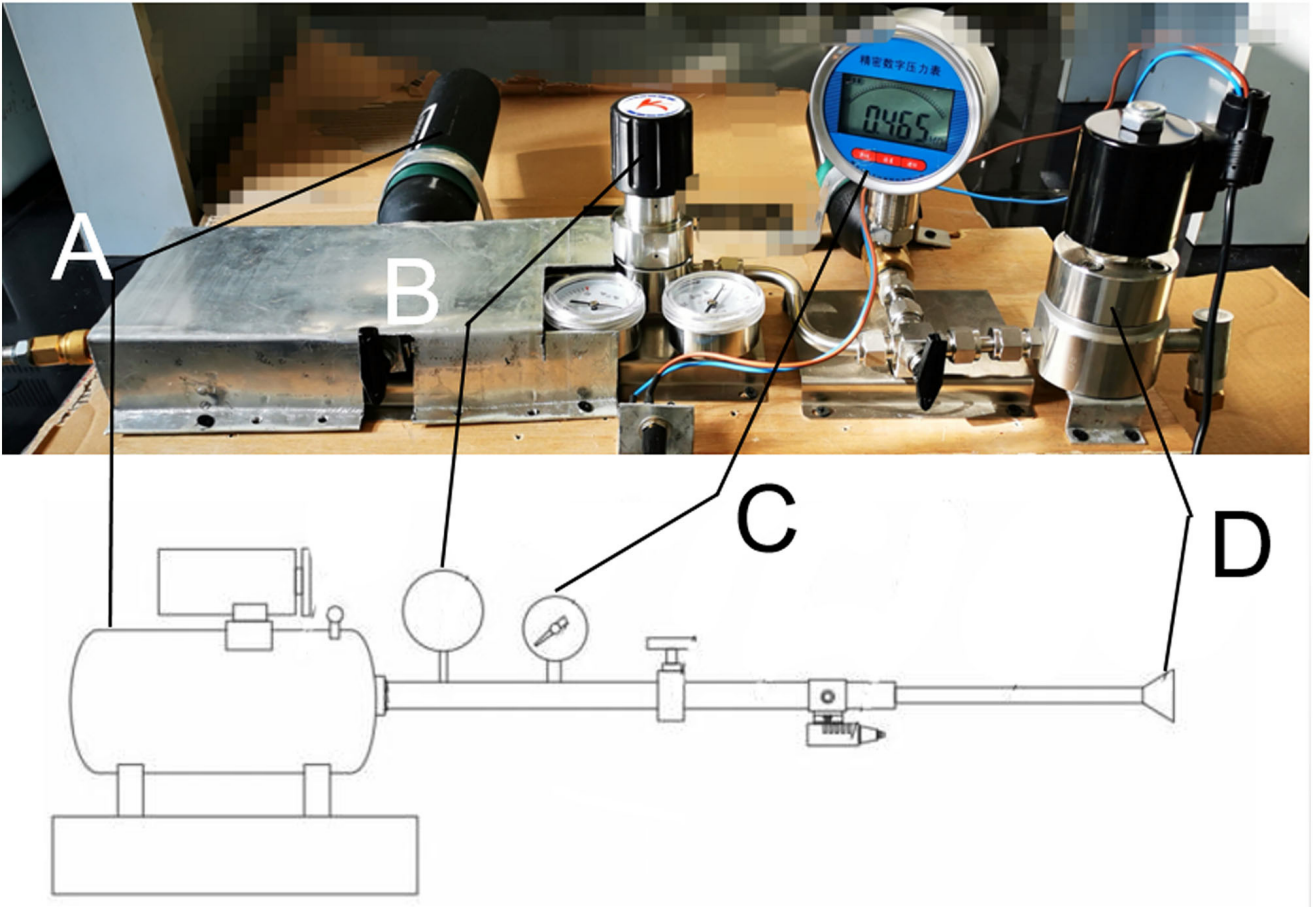
Picture of the real blast device corresponding to the schematic diagram. **(A)** High-pressure air source generating device. **(B)** Pressure regulating valve. **(C)** Piezometer. **(D)** Nozzle.

The device used in this study was self-designed with patent approved (201920642373.7), which was successively connected by a high-pressure air source compression pump, air flow pipeline, and gas injection unit (Figure 1). The output section of the pump is sealed with the port of the connecting flange and the airflow pipeline followed by connection to the gas pressure regulators, the valve, and gas pressure measuring device in turns, and the part of the jet gas is composed of an integrated connecting pipe and nozzle. The high-pressure gas compression pump compresses the air in the air storage tank, and the flow of gas in the pipeline was controlled *via* opening or closing valve. The pressure of the gas is adjusted by the pressure regulating part. High-pressure gas flowed through the air storage, pressure regulating part, air flow pipe, and connecting pipe, then, the gas was injected to the eye of the rabbit through the nozzle. By compressing the high-pressure gas to the gas storage tank with the gas pressure adjusted according to the indicator, the high-speed solenoid valve controls the switch of the jet nozzle to spray the high-pressure gas that can simulate the blast eye injury.

### Closed Globe Blast Injury

In this study, 36 rabbits were divided into six groups according to the different blast pressures imposed evenly (*n* = 6): blank control, 500 Kpa group, 1,000 Kpa group, 1,500 Kpa group, 2,000 Kpa group, and 5,000 Kpa group. The rabbits were starved for 12 h and anesthetized with chloral hydrate (3.5 ml/Kg) for general, followed by Obrucaine hydrochloride eye drops for topic anesthetization. Left eye of the rabbit was fixed 0.5 cm from the nozzle, followed by adjustment of blast pressure to the predicted value. Then, a high speed-solenoid valve switch was turned on, and the corneal center was exposed to the shock wave, hitherto, the closed globe blast injury was created by the blunt block. The antibiotic eye drops were used for the injured eyes four times a day for 3 days.

### Eye Examination

The eyes of all the rabbits' pre- and post-injury were carefully examined by slit lamp and fundus scope with photographs of anterior and posterior segments collected. The abnormality of the eyes was recorded thoroughly.

### Measurement of Intraocular Pressure

Intraocular pressure (IOP) of the rabbit animal model were measured pre-injury and at 0, 1, 3, 7 days post-injury using a tonometer (NIDEK, JAPAN).

### Optical Coherence Tomography

Rabbits were anesthetized as above, with sufficient pupil dilation. Then, the head of the rabbits was fixed to the holder of the optical coherence tomography (OCT) scan machine. The retinas were thoroughly imaged using the OCT system from the optic disc to the peripheral retina as much as possible. Representative images were presented in the Results Section.

### B Ultrasound Scan

The rabbits were anesthetized as above and wrapped to keep warm. The probes of the ultrasound machine went through the eyes in turns to observe the position of the lens, vitreous status, papilledema, retinal, and choroidal changes. Typical images with positive changes were illustrated in the Results Section.

### Eye Histopathology

The entire eye was carefully enucleated with part of the optic nerve left 7 days post-injury. Then, the eye was fixed, dehydrated, and embedded in sequence. Afterward, the slices were cut and dyed with hematoxylin-eosin to observe the tissue pathology in detail.

### Statistics

A statistical analysis was performed by SPSS 26.0 (SPSS Inc., Chicago, IL, USA). The values of IOP were described as Mean ± SE. The values of IOP in each group at different pressure were analyzed *via* ANOVA and the least significant difference (LSD) *post-hoc* test for different time points at the same pressure. When the distribution does not conform to the normal distribution, data were described as median (interquartile interval), and the rank sum test was used to analyze the differences. *P*-value < 0.05 was considered of statistical significance.

## Results

### Corneal Edema and Hyphema Occurred After Blast Injury With No Laceration or Globe Rupture

Corneal edema and hyphema were observed in all eyes induced by gas shock at all pressure, although the percentage of that was much higher in groups of higher pressure (≥1,000 Kpa). However, no iris laceration, lens rupture, or lens dislocation were observed in any of the groups.

**Figure 2:**
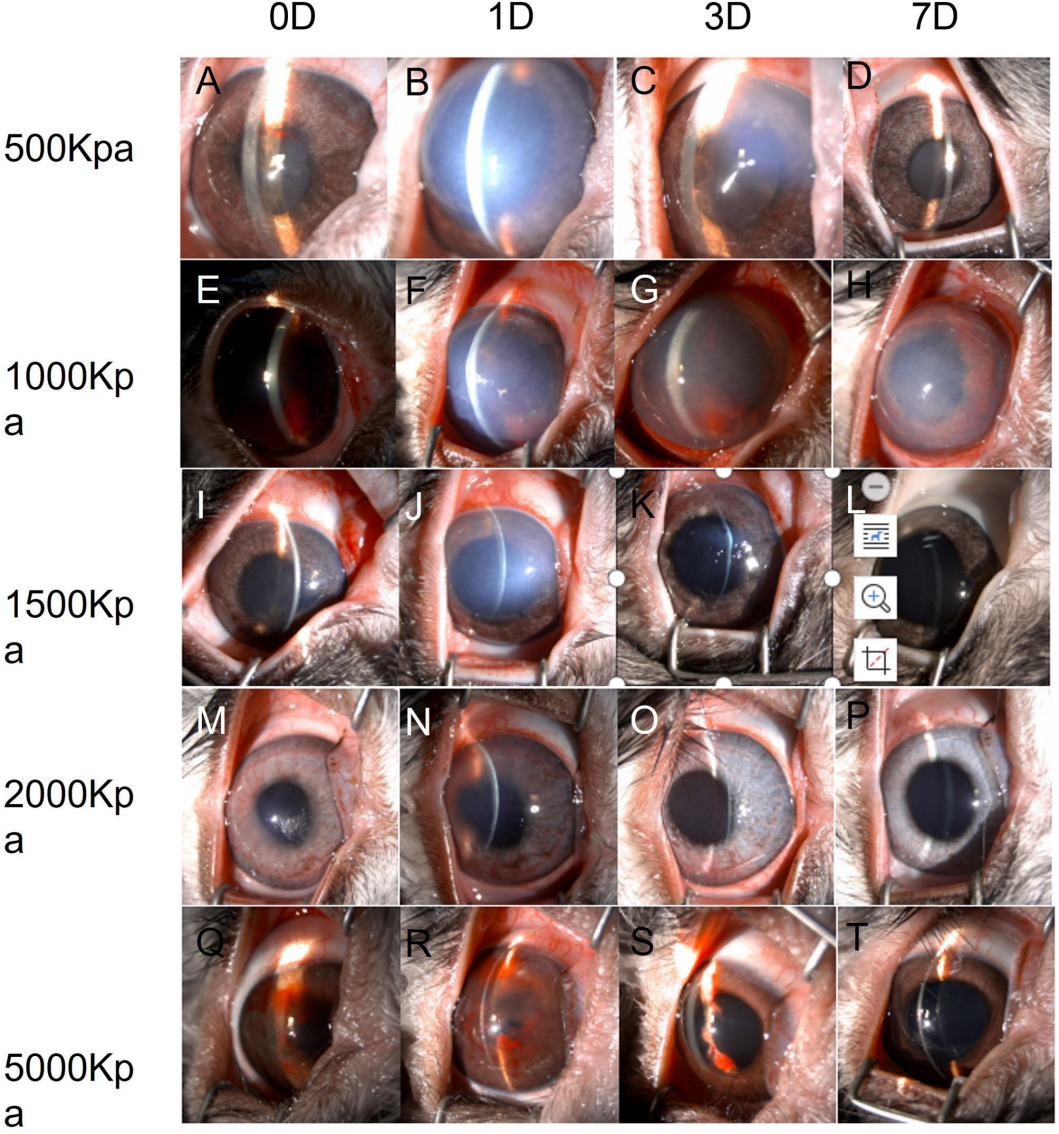
Changes of eyes exposed to different pressure examined by slit lamp chronologically. **(A–D)**: Changes of eyes exposed to 500 Kpa. **(A)** Minor hyphema combined with corneal lesion on day 0. **(B)** Corneal opacity at day 1 **(C)** Corneal edema was ameliorated at day 3. **(D)** Transparent cornea and anterior chamber at day 7. **(E–H)**: Changes of eyes exposed to 1,000 Kpa. **(E)** Moderate hyphema combined with corneal lesion on day 0. **(F)** Corneal opacity at day 1. **(G)** Corneal edema was ameliorated, and blood-fluid level occurred at day 3. **(H)** Slightly residual cornea edema and clear anterior chamber with irregularly dilated pupil at day 7. **(I–L)** Changes of eyes exposed to 1,500 Kpa. I. Minor corneal edema on day 0. **(J)** Moderate corneal opacity at day 1. **(K)** Transparent corneal with dilated pupil at day 3. **(L)** Transparent corneal with dilated pupil at day 7. **(M–P)** Changes of eyes exposed to 2,000 Kpa. **(M)** Minor corneal lesion with scattered hyphema on day 0. **(N)** Moderate corneal lesion with lightly scattered hyphema at day 1. **(O)** Minor corneal edema at day 3. **(P)** Transparent corneal at day 7. **(R–T)** Changes of eyes exposed to 5,000 Kpa. **(Q)** Minor corneal lesion with severe hyphema on day 0. **(R)** Enlarged corneal edema with hyphema attached to the corneal endothelium at day 1. S. Minor corneal edema at day 3 with residual hyphema. **(T)** Transparent corneal with dilated pupil at day 7.

**Figure 3:**
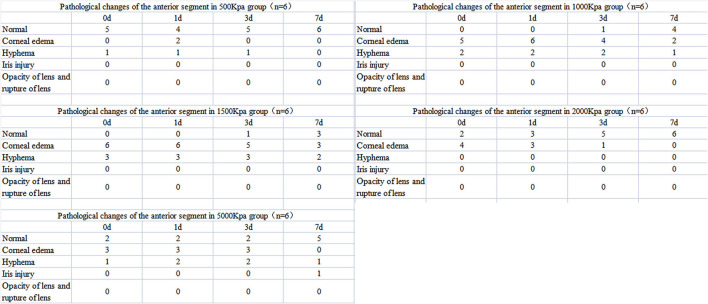
Summary of pathological changes of anterior segment in eyes exposed to all pressure.

In 500 Kpa group, four out of six rabbits did not show any obvious anterior segment damage, while the two rabbits (33.3%) showed corneal edema 1 day post-injury with one accompanied with hyphema on the day of injury. All the above signs disappeared on day 7 post-injury (Figures 2A–D, 3).

In 1,000 Kpa group, corneal edema occurred on five injured eyes and hyphema occurred on two injured eyes, none of them were recovered by 1 day post-blast. One of the rabbit models recovered completely on day 3, and three recovered on day 7 of post-blast (66.66%) (Figures 2E–H, 3).

In 1,500 Kpa group, corneal edema occurred on all the injured eyes with three combined with hyphema, which were more severe in appearance compared with the other groups. On day 1 post-injury, the symptoms of one eye were relieved and the rest were accelerated. One of the injured eye recovered from the entire damaged signs on day 3, while the other two eyes were recovered on day 7 (50%) (Figures 2I–L, 3).

In 2000 Kpa group, corneal edema occurred on four injured eyes, whereas no hyphema occurred. On day 7, all the above eyes had transparent cornea, normal iris, and no infiltration or hyphema (one on day 1 and two on day 3) (Figures 2M–P, 3).

In 5,000 Kpa group, corneal edema occurred on three injured eyes and hyphema occurred on one injured eye on the day of blast, one of which was recovered on day 1 and three were relieved to normal on day 7 (80%). Additionally, one eye presented corneal edema and hyphema on day 1, in which hyphema were organized to definite membrane overlapping the lens and posterior synechia of the iris occurred on day 7 (Figures 2Q–T, 3).

In total, there were 22 rabbit models that showed obvious damages of the anterior segment at four time points, with no open injury of cornea, opacity of lens, or rupture of lens were observed. There was no significant correlation between the severity of anterior segment damage and gas pressure. Finally, 16 cases of rabbit models were self-healed without any interventions (Figure 3).

### Slight Vitreous Hemorrhage and Commotio Retinae Induced by 5,000 Kpa Gas Pressure

**Figure 4:**
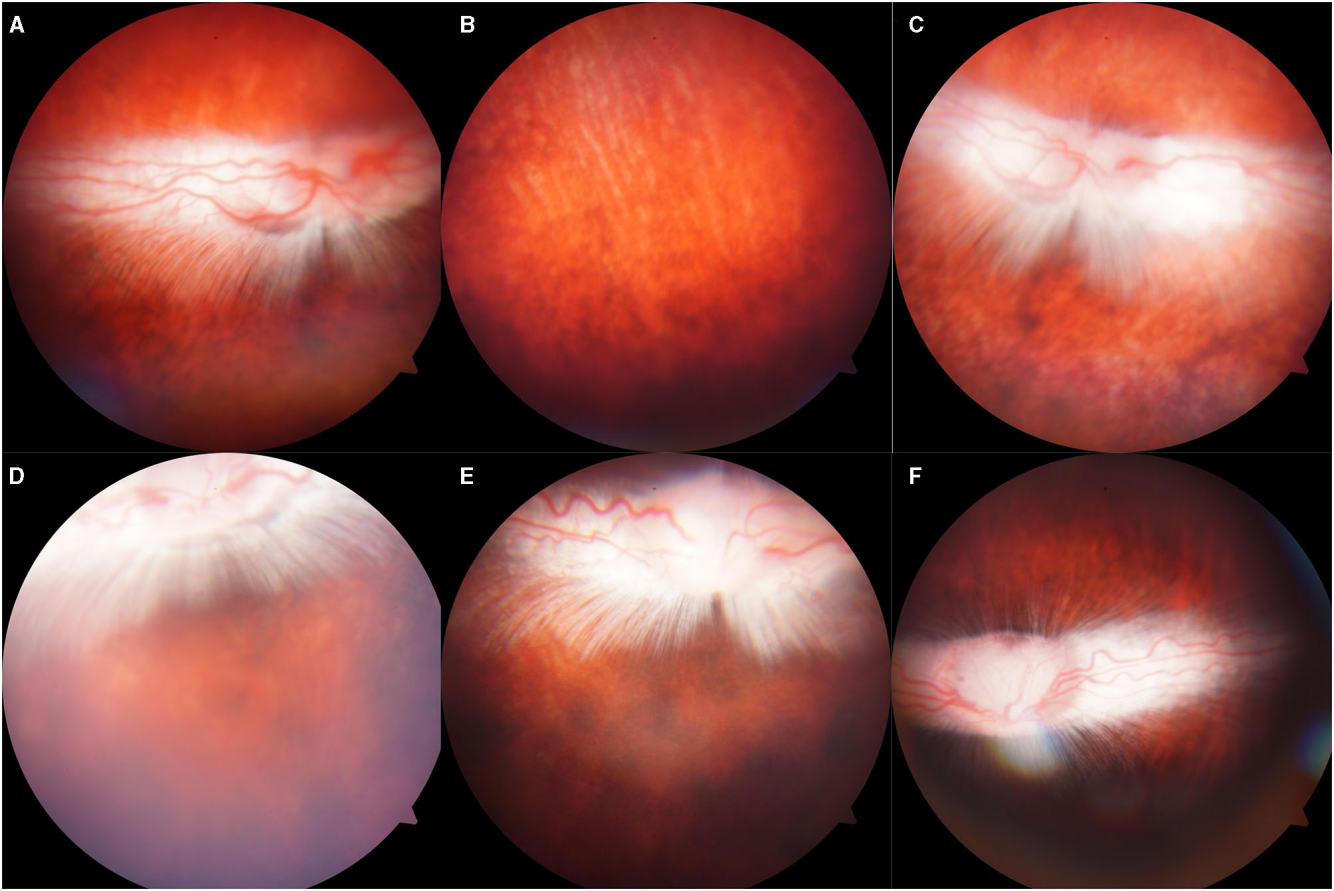
Representative fundus photographs obtained from the eyes of the rabbits. **(A,B)**: Normal group: Retina was well-attached with clear vessels. **(C–F)**: Eyes exposed to 500, 1,000, 1,500, 2,000: Retina was attached, no retinal bleeding or vitreous hemorrhage was detected.

**Figure 5:**
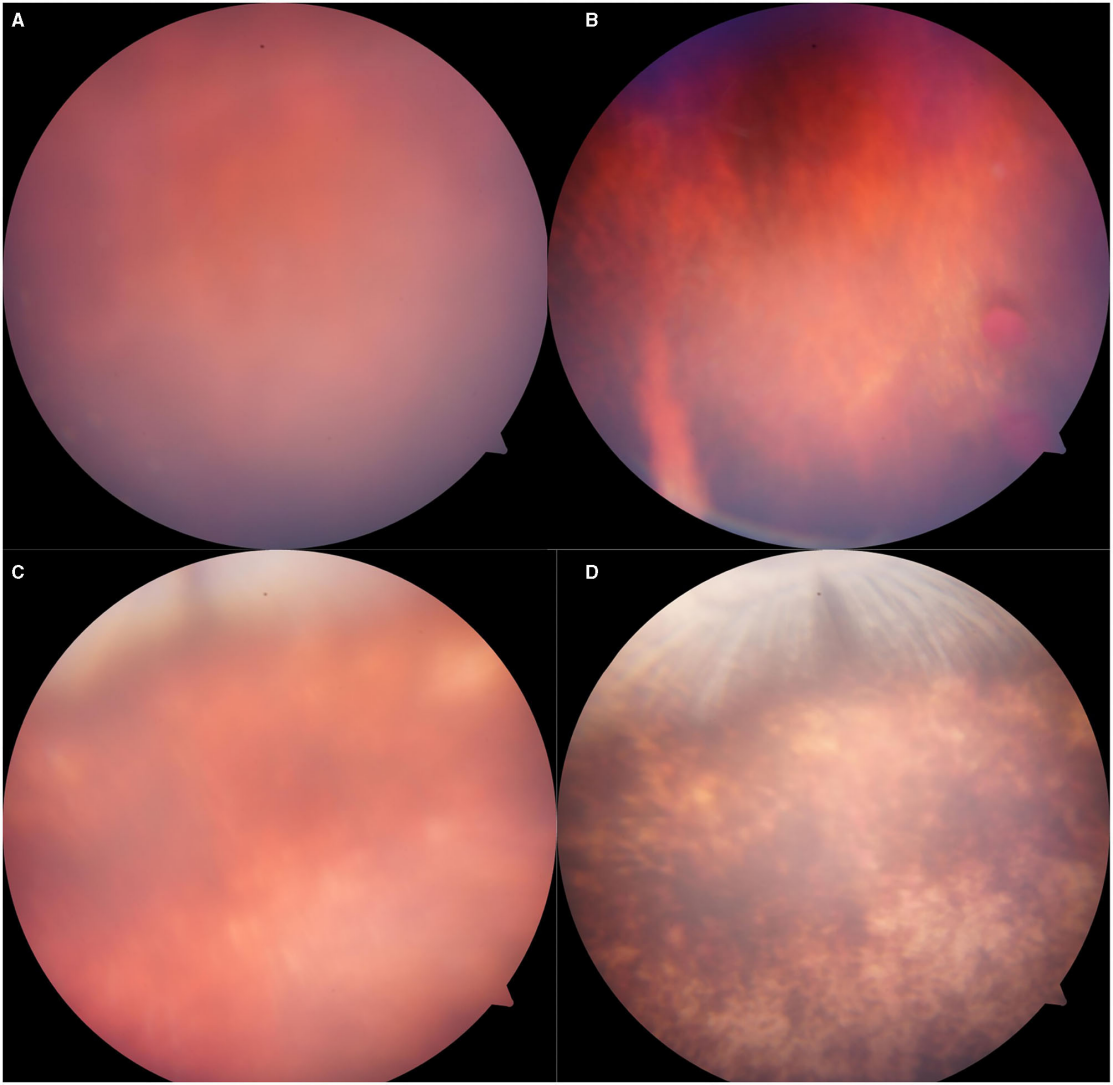
Representative fundus photographs obtained from the eyes of rabbits exposed to 5,000 Kpa. **(A)** Fuzzy fundus at day 3. **(B)** Minor vitreous hemorrhage. **(C)** Blurred fundus with suspected commotio retinae at day 3. **(D)** A majority of retinal pigmentation.

There was abnormality shown on the fundus photograph of the control group: transparent vitreous, clear edge of optic disc, normal vessels, and attached retina (Figure 4). Except for the blurred fundus due to corneal opacity, no vitreous hemorrhage or opacity, retinal tear, retinal hemorrhage, or retinal detachment occurred in the rabbit models induced by pressure <2,000 Kpa (Figure 4). In rabbit animal models induced by gas shock under 5,000 Kpa: one had slight vitreous hemorrhage without retinal detachment on day 7 (Figure 5); another one presented fuzzy fundus appeared as orange due to the severe corneal edema on day 3, which tended to brown yellow with substantial pigmentation and no retinal detachment indicating commotio retinae occurred previously (Figure 5).

**Figure 6:**
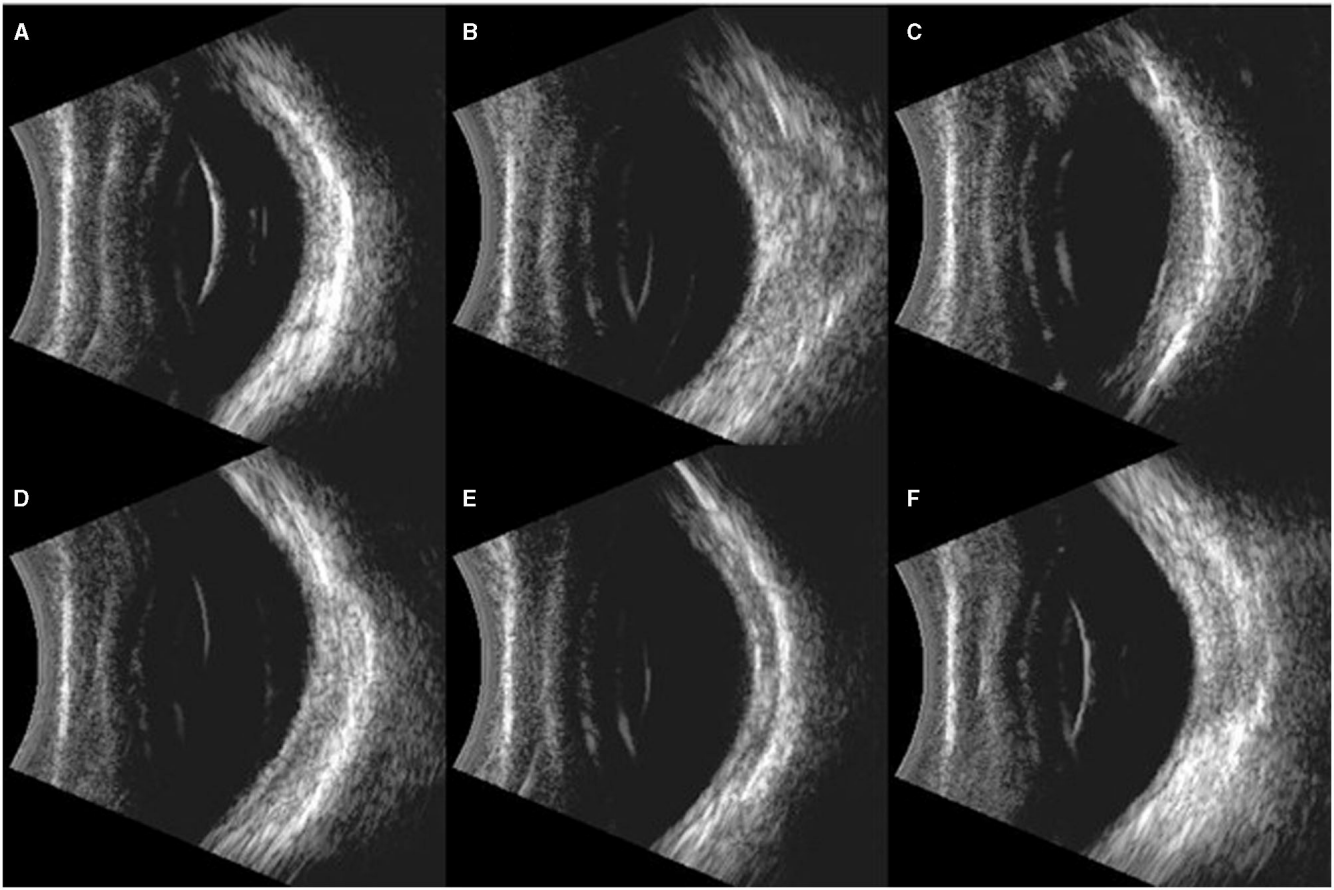
Typical images of B ultrasound scan on the eyes of rabbits. **(A)**: Blank control group: Retina was well-attached with clear vitreous. **(B–F)**: Eyes exposed to 500, 1,000, 1,500, 2,000, and 5,000 Kpa: Retina was well attached, and no vitreous lumpy echo was detected.

**Figure 7:**
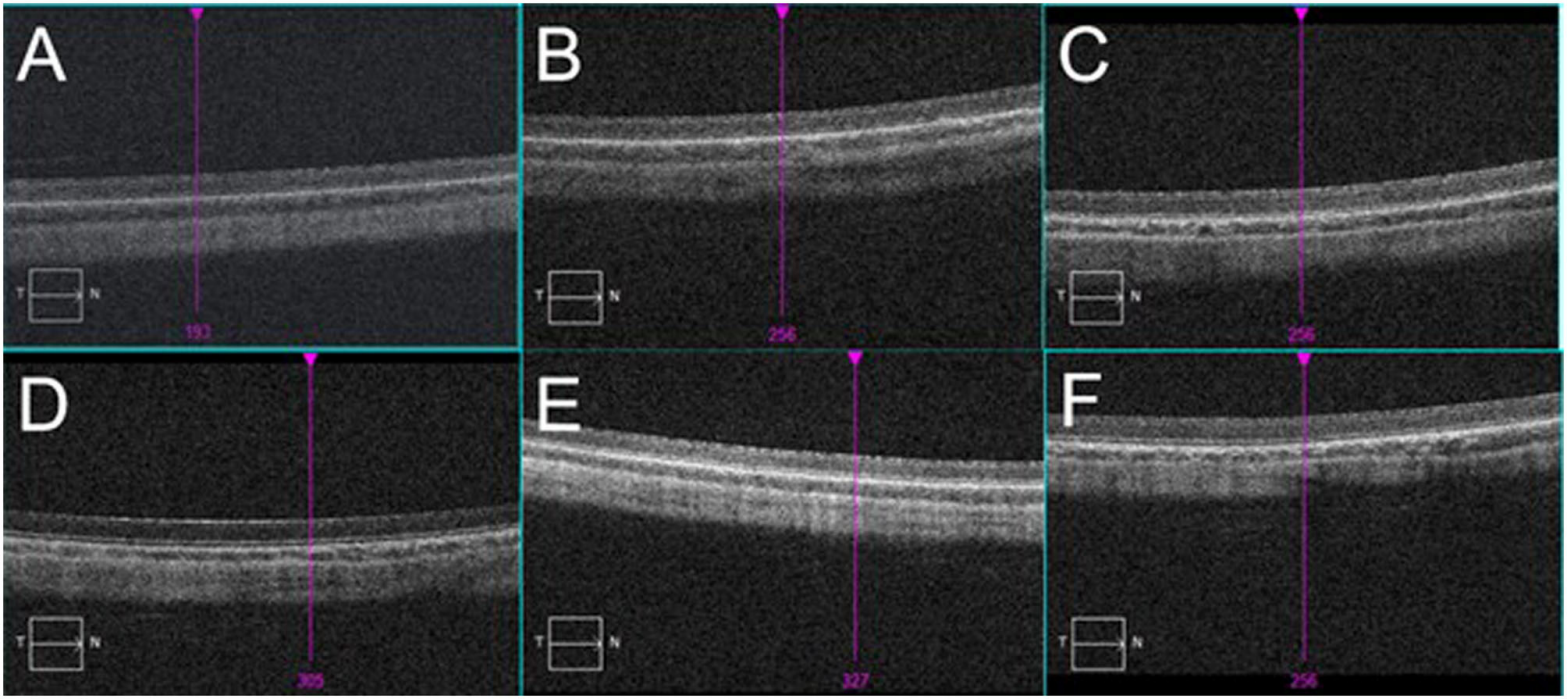
Typical images of OCT scan on the eyes of rabbits. **(A)**: Blank control group: retina was well-attached, and layers of retina were in order. **(B–F)**: Eyes exposed to 500, 1,000, 1,500, 2,000, and 5,000 Kpa: No abnormality was observed.

Compared with the pre-blast rabbits of experimental groups and control group, none of the injured eyes showed vitreous strong echo or retinal detachment 7 days post-blast (Figure 6). Similarly, all retinas on the injured eyes were attached with distinct structures of each layer stood out on the OCT scans on day 7 (Figure 7).

### Increased Retinal Thickness and RGC Apoptosis Induced by Gas Shock at Higher Pressure

**Figure 8:**
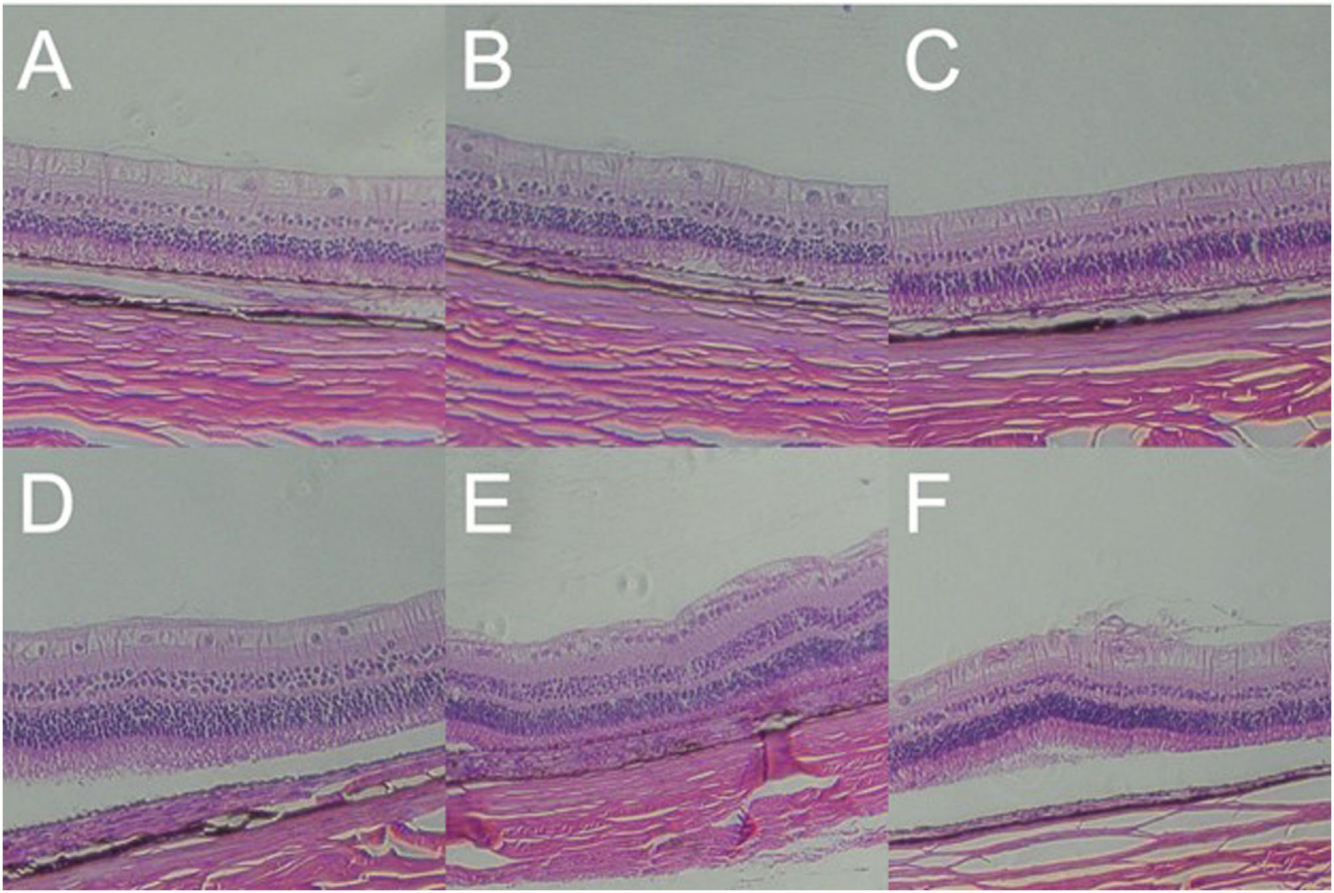
Gross pathology of retinas of animal model with closed globe blast injury at day 7. **(A)**: Blank control group: 10 layers of the retina were integral and well-organized with normal thickness. Morphology of retinal cells were normal (× 100). **(B)** 500 Kpa group: Retinal ganglion cells (RGCs) were arranged in order, and the nucleus were distributed evenly (× 100). **(C)** 1,000 Kpa group: The retinal ganglion cells were arranged neatly, and the nucleus arrangement was tighter than control group (× 100). **(D)** 1,500 Kpa group: The nucleus arrangement was tighter than 1,000 Kpa group (× 100). **(E)** 2,000 Kpa group: The retinal ganglion cells were disordered, and the nucleus were dense (× 100). **(F)** 5,000 Kpa group: RGCs nucleus were karyopyknotic, and like vesicular nucleus (× 100).

A histopathological test showed that the retinal layers of the blank control group were intact with normal structure and morphology, and the cells were arranged neatly and compactly. However, in 1,000, 2,000, and 5,000 Kpa group, the retinas of the rabbit animal model were thickened, and the retinal ganglion cell layer (RGCL) were arranged disorderly, and the nucleus of RGC was packed densely. Moreover, the retinal nucleus developed to pyknosis gradually as the increase of gas pressure was exposed to rabbit eyes at day 7 post-blast, and the arrangement of RGCL also tended to be more disordered accompanied by a patch of tear in the IS/OS layer. The retinal structure and cells in rabbits exposed to 500 Kpa gas shock were neatly arranged compared with other gas pressure groups, which was similar to that of the blank control group (Figure 8).

### Blast Injury Caused Inflammatory Cells Infiltration in Cornea and Stroma Damage

**Figure 9:**
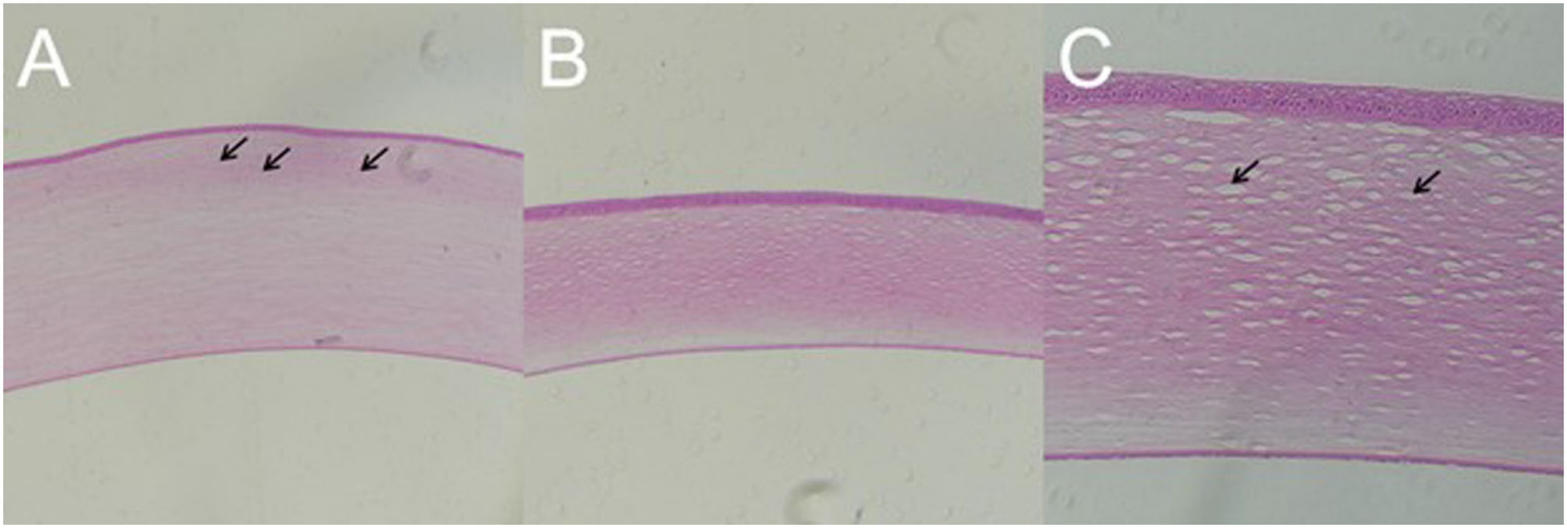
Gross pathology of cornea of animal model with closed globe blast injury at day 7. **(A)**: Eyes with residual corneal edema: The stroma layer was thickened, where the inflammatory cells infiltrated close to the external elastic layer shown by black arrows (× 100). **(B,C)**: Corneal edema occurred immediately after blast and self-healed at day 7: Stromal thickness was normal (× 100), whereas loosen stroma was left at higher magnification shown by black arrow (× 200).

In all the established rabbit models, there was no significant change in the corneal epithelial cell layer and endothelial cell layer on day 7 after blast exposure. However, in the rabbit with residual corneal edema, the stroma layer was thickened obviously, where the inflammatory cells infiltrated close to the external elastic layer (Figure 9A). Corneal histology of rabbit eye with corneal edema immediately after blast exposure and receded at day 7 showed that the thickness of corneal stroma layer was basically normal (Figure 9B). Under the observation of higher magnification, the tissue of stroma layer was loose, whereas the structure of the epithelial cell layer and endothelial cell layer was still intact (Figure 9C).

### IOP Decrease Was Independent of Blast Pressure and Injury Duration

**Figure 10:**
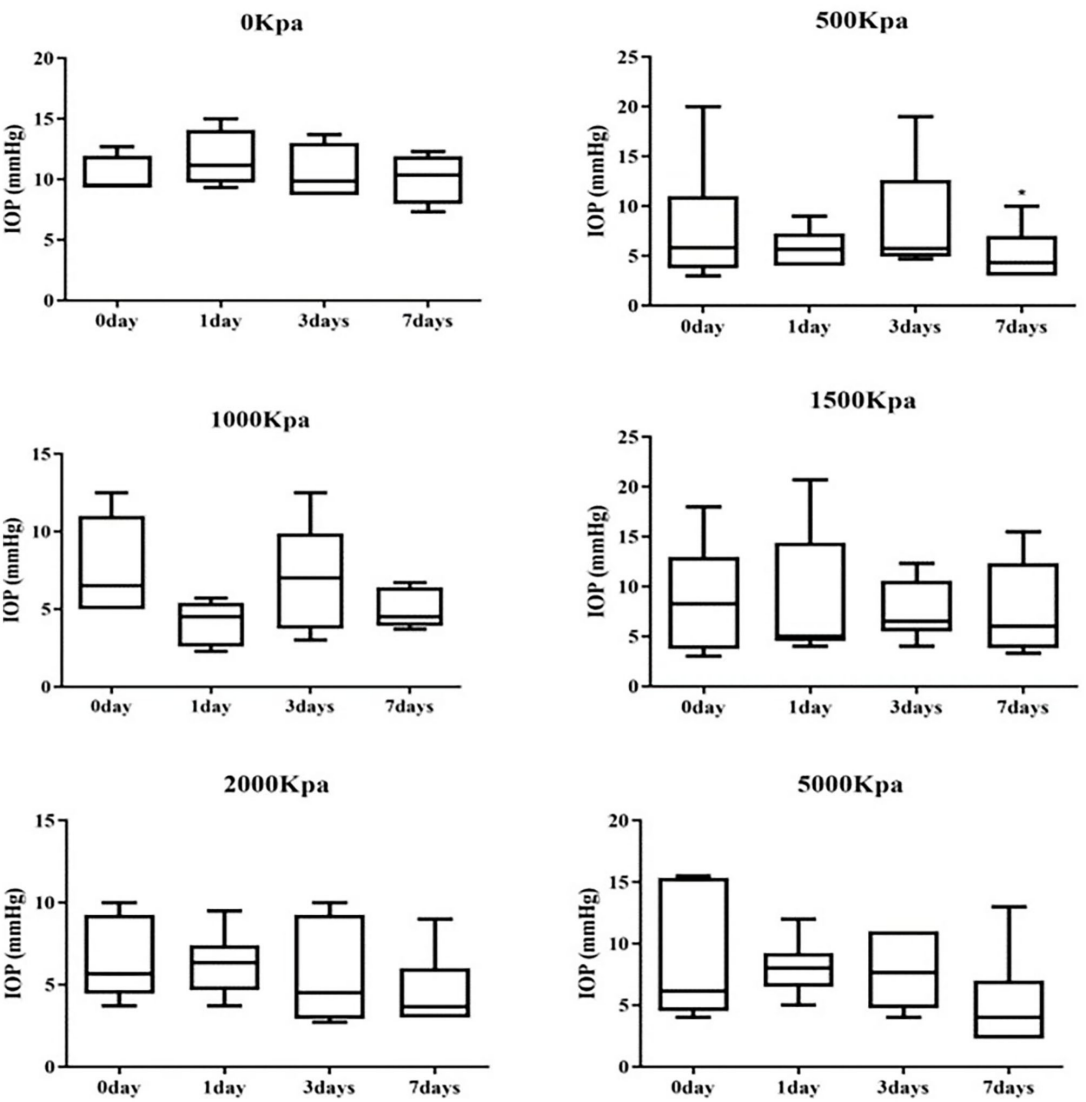
Whisker plot of intraocular pressure (IOP) measured at 0-, 1-, 3-, and 7-days post-blast. ^*^Means *p* < 0.05 compared with day 3.

**Figure 11:**
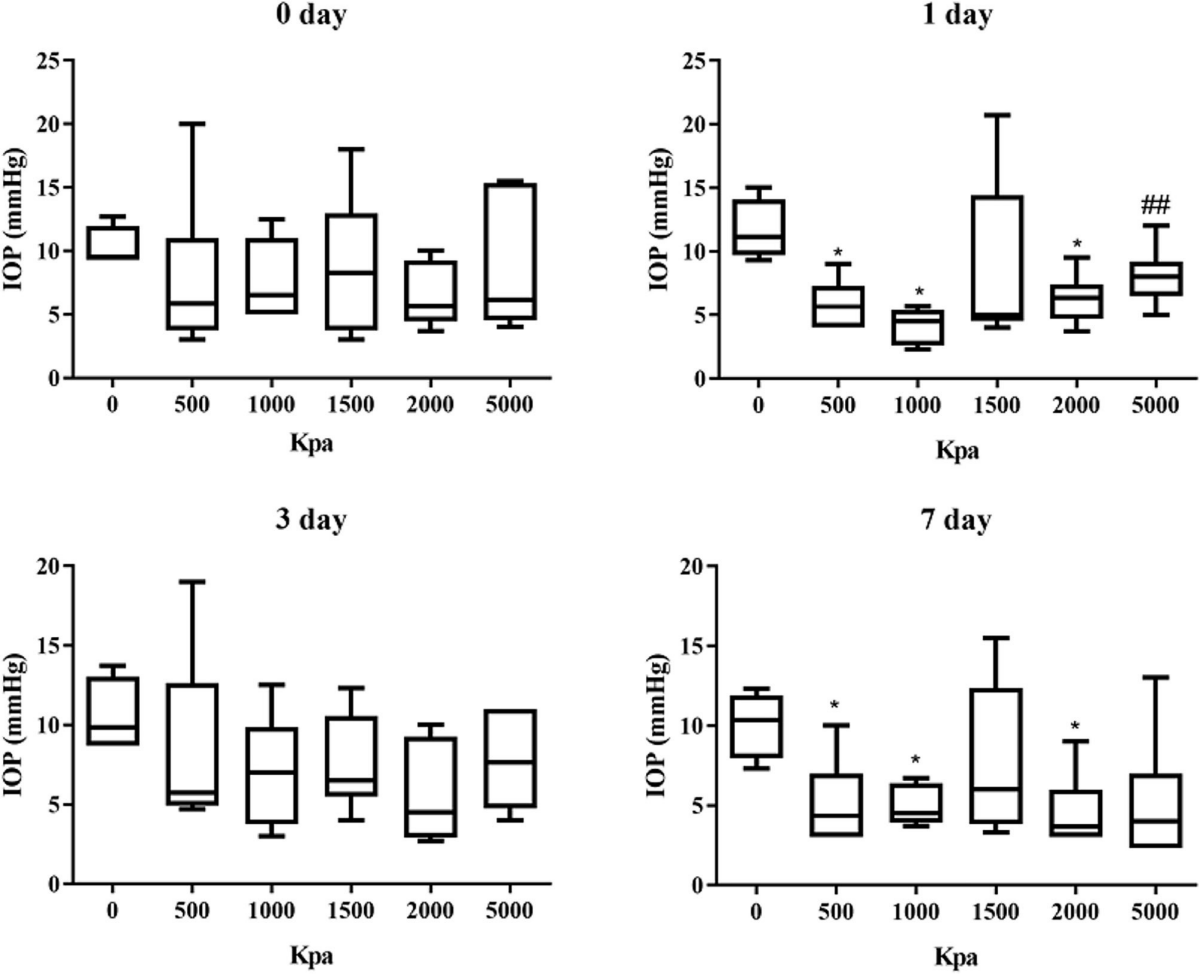
Graphs of changes of IOP with gas pressure detected at the same time point. ^*^Means *p* < 0.05 compared with the blank control group. ##means compared to the 1000 Kpa, the decrease of IOP in eyes exposed to 5000 Kpa was statistically significant with *p* < 0.05.

The IOP of the rabbits' eyes received gas shock under 500, 1,000, 2,000, and 5,000 Kpa were decreased significantly on day 1 and 7 post-blast (*p* < 0.05). The IOP of other groups detected at the specific time point were not statistically significant despite a minor increase or decrease within 5 mmHg. Longitudinally, there was no clear correlation between the change of IOP and the injured time (Table 1). In addition, changes of IOP gas pressure-independent statistically with *P* > 0.05 (Figures 10, 11).

**Table 1:** Values of IOP in different groups (Median and IOR).

	**0 days**	**1 day**	***P*** ^**a**^	**3 days**	***P*** ^**a**^	***P*** ^**b**^	**7 day**	***P*** ^**a**^	***P*** ^**b**^	***P*** ^**c**^
对照组	9.50 (9.30, 11.95)	11.15 (9.73, 14.07)	0.144	9.85 (8.70, 13.02)	0.715	0.465	10.35 (7.97, 11.90)	1.000	0.068	0.461
500 KPa	5.85 (3.75, 11.00)	5.65 (4.00, 7.27)	0.916	5.75 (4.92, 12.62)	0.833	0.116	4.35 (3.00, 7.00)	0.462	0.400	0.043
*P*_1_	0.087	0.010		0.199			0.024			
1,000 KPa	6.50 (5.00, 11.00)	4.50 (2.60, 5.40)	0.046	7.00 (3.75, 9.87)	0.674	0.043	4.50 (3.92, 6.40)	0.058	0.498	0.176
*P*_1_	0.257	0.010		0.171			0.010			
*P*_2_	0.589	0.180		0.937			0.699			
1,500 KPa	8.25 (3.75, 12.98)	5.00 (4.52, 14.40)	0.916	6.50 (5.50, 10.57)	0.345	0.752	6.00 (3.82, 12.35)	0.500	0.752	0.686
*P*_1_	0.352	0.352		0.171			0.352			
*P*_2_	0.699	0.818		0.699			0.240			
*P*_3_	0.937	0.310		0.937			0.394			
2,000 KPa	5.65 (4.45, 9.25)	6.35 (4.67, 7.40)	0.753	4.50 (2.92, 9.25)	0.462	0.674	3.65 (3.00, 6.00)	0.248	0.080	0.080
*P*_1_	0.067	0.019		0.114			0.019			
*P*_2_	0.937	0.699		0.132			0.699			
*P*_3_	0.394	0.065		0.589			0.310			
*P*_4_	0.589	0.937		0.240			0.132			
5,000 KPa	6.15 (4.53, 15.35)	8.00 (6.50, 9.22)	0.917	7.65 (4.75, 11.00)	0.917	0.916	4.00 (2.30, 7.00)	0.249	0.207	0.225
*P*_1_	0.476	0.067		0.352			0.114			
*P*_2_	0.818	0.093		0.937			0.699			
*P*_3_	0.937	0.009		0.699			0.485			
*P*_4_	0.937	0.485		0.937			0.240			
*P*_5_	0.699	0.132		0.132			0.937			

*P^a^ compare with 0 days*,

*P^b^ compare with 1 day*,

*P^c^ compare with 3 days*.

*P_1_, means compare with control group; P_2_, means compare with 500 KPa group; P_3_, means compare with 1,000 KPa group; P_4_, means compare with 1,500 KPa group; P_5_, means compare with 5,000 KPa group*.

## Discussion

In this study, we successfully established the reproducible rabbit animal model of closed globe blast injury induced by self-developed instruments under an optimized condition similar to eye damage reported in the survivors of the war or civilian explosion, especially for the anterior segment (7, 16). Although there were no clinical signs for the retinal changes, definite retinal thickening and apoptosis were detected. On behalf of the development of a stable animal model, the gas shock produced by the device used is pressure exposed in a controllable and measurable manner by altering the input pressure of the tank. The gas was well-accumulated to the eyes exposed, hitherto, the contralateral eye and other parts of the rabbit body, especially the brain, were not affected during the blast. Superior to the other systems used for the same animal model as reported (10, 17), there was no mortality or open globe injury detected in this study.

Previously, the rodents (C57Bl/6J and DBA/2J and Balb/c mouse strains or rats) were mostly used for the animal model with low costs, whereas less tissue was obtained and higher lethality (18). Additionally, scaling principles have been achieved that injury biomechanical effect caused by blast explosion may differ significantly based on eye size and animal size (19). Although enucleated porcine eyes were also used to overcome the limitations of rodents, the ocular pathological changes could not be observed longitudinally (20). In that case, rabbits with closer anatomical similarities to the human eyes and larger size of eyes were chosen for this study (21). Airflow extruded by the gas shock was dispersed evenly on the rabbit's eyes without affecting the organs close to the eyes (9). Corneal edema, hyphema, and thickened retinal thickness were detected in the rabbit animal model, which might be relevant to clinical vision loss in patients who suffered ocular blast injury.

We set up a gradient of pressure ranging from 500 to 5,000 Kpa to find out the optimum pressure for the blast. Successful rate of the rabbit animal model in each experiment group was 33.3%, 100%, 100%, 66.7%, 83.3%, and in total was 76.7%, which was much higher than the previous animal models reported (30%) (9). There were identical pathological changes on the anterior segment that were commonly found in rabbits' animal models right after the blast exposure. Consistent with the patients or animal models reported, corneal edema occurred within 24 h post-blast was the most common damage (7, 22) due to a sharp decrease of the endothelial cells density, the rate of which in our model was much higher than the one reported by Jones K (23). The majority of corneal edema was receded on day 7, despite the corneal stroma was loosen. The prolonged corneal edema indicated that the dysfunction of endothelial cells in the cornea induced by the blast injury and inflammation shown with inflammatory cells infiltration close to extra elastic layer (23). As Daniel reported, inflammatory factors, such as IL-1b and LIX were elevated between day 1 and 4 weeks after the blast (10), and LIX was associated previously with neutrophil infiltration to the stroma and keratitis (24). The self-healing of corneal edema 7 days post-blast was attributed to the integrity of corneal endothelium shown on the histology slices, since the endothelial cells in rabbits can be regenerated (25), which could be proved by confocal microscopy and Endothelial cell count in the further study.

Clinically, hyphema was a vital sign of iris damage, which occurred frequently in blast induced globe injury (8). However, the present animal models rarely focused on the observation of hyphema after blast (26). In our rabbit animal model, hyphema occurred in eight rabbits, especially in 1,500 Kpa group, within 24 h post blast indicating blunt damage to the vessels of the iris. Hyphema in five out of eight rabbits were absorbed without any interventions at day 7 post blast, while the rest were sustained, even to anterior synechia in rabbit, the rate of which conformed to the clinal data collected in veterans (7). Self-absorption of hyphema was relied on less amount of blood and healthy anterior chamber angle. Persistent blood accumulated indicated damage or obstruction to the anterior chamber angle, which could result in the elevation of IOP followed by optic nerve damage, especially in the older patients (27). Moreover, retrospective study about traumatic hyphema from combat ocular injury recorded in Walter Reed Ocular Trauma Database (WRTOD) demonstrated that traumatic hyphema were highly associated with the traumatic cataract formation, retinal detachment, angle recession, and final VA of <20/200 (28). Although none of the rabbit model showed cataract, which may come out as observation prolonged.

The changes of IOP about present animal models of ocular blast injury were controversial since multiple factors could have impacts on IOP oppositely. Generally, ketamine anesthesia is proven to elevate IOP, while isoflurane is proven to suppress it (29). Locally, corneal edema, function of ciliary body, and anterior chamber recession may also affect the IOP. Additionally, IOP changes were controversial in different strains of mice, which were elevated in the Balb/c postinjury and decreased in the C57Bl/6J. The reasons could be substantial corneal injuries that occurred in the Balb/c and a spot of corneal injuries in the C57Bl/6J post injury (18). Consistent to the studies of Jessica Hines-Beard and Kirstin Jones, IOP were significantly decreased in rabbits of 500, 1,000, and 2,000 groups at day 1 and day 7 post blast, which was independent of the exposed pressure. The IOP of the rest groups were increased or decreased within 5 mmHg, although there was no statistical significance with this variation. The IOP elevation was attributed to outflow block caused by anterior chamber recession or obstruction, whereas the IOP decrease was due to ciliary muscle dysfunction or corneal edema with a slight negative trend to the IOP measurement (30). IOP changes could be suggestive of traumatic glaucoma, but more improvements should be achieved on the IOP measurement instrument and anterior chamber test with Ultrasound Biomicroscope (UBM) or gonioscope or anterior OCT.

In rabbits exposed to 5,000 Kpa, only one rabbit presented minor vitreous hemorrhage, and another one presented potential commotio retinae with retinal pigmentation left at day 7. There were no obvious clinical signs of increased retinal thickness or detachment on the rest of the rabbit animal models, which was close to the results of the study of Shedd (10). However, there were definite retinal thickening and RGC apoptosis on the retinal sections of eyes exposed to 5,000 Kpa, which indicated that the higher pressure could reach the retina pass through the anterior chamber causing retinal damage proven by the research of Jones (23, 31). Since the retinal thinning and neuron degeneration were observed varied from 2 weeks to 3 months post-injury as literature reported (32). We assume that there will be retinal damages, such as retinal thinning and RGC degeneration will occur in this animal model when the observation time is extended. In addition, electroretinography (ERG)or visual evoked potentials (VEP) could be added to reveal the retinal function as a complement to the structural changes.

Apparently, this study has demonstrated that primary blast itself could induce closed globe injury (2), but the severity of ocular damage is relatively lower for the patients in combats or civilian blast injury (8). Future study may focus on bringing in explosive substances to our blast device on behalf of creating an animal model with blast eye injury closer to the clinics.

In conclusion, we established the rabbit animal model of closed globe blast injury induced by self-developed explosive device under optimized pressure. Eyes exposed to 1,500 Kpa could be an ideal model for traumatic corneal damage and hyphema, whereas the eyes exposed to 5,000 Kpa could serve as a model for traumatic retinal damage. Our research can provide a platform to reveal the underlying mechanism of blast eye injury and verify the new therapeutic interventions preclinically.

## Data Availability Statement

The original contributions presented in the study are included in the article/supplementary material, further inquiries can be directed to the corresponding author/s.

## Ethics Statement

The animal study was reviewed and approved by Institute of Radiation Medicine, Chinese Academy of Medical Sciences.

## Author Contributions

YL, TY, JY, and HY designed this study. YL, TY, ML, and JL collected and measured data. YL and TY analyzed, interpreted the dataset and wrote this article. HY revised the manuscript. All authors contributed to the article and approved the submitted version.

## Funding

This study was supported by National Natural Science Foundation of China (Grant Numbers 81900883, 82020108007) and Tianjin Natural Science Foundation (Grant Number 19JCQNJC11300). The sponsor or funding organizations had no role in the design or conduct of this research.

## Conflict of Interest

The authors declare that the research was conducted in the absence of any commercial or financial relationships that could be construed as a potential conflict of interest.

## Publisher's Note

All claims expressed in this article are solely those of the authors and do not necessarily represent those of their affiliated organizations, or those of the publisher, the editors and the reviewers. Any product that may be evaluated in this article, or claim that may be made by its manufacturer, is not guaranteed or endorsed by the publisher.
